# 西达本胺联合R2-CHOP方案治疗复发难治EZB/C3亚型弥漫大B细胞淋巴瘤一例

**DOI:** 10.3760/cma.j.issn.0253-2727.2021.05.016

**Published:** 2021-05

**Authors:** 理 杨, 炜 张, 美兰 张, 克锋 沈, 佳晨 汪, 玉琪 关, 浩东 蔡, 毓函 包, 敏 肖, 剑峰 周, 邈 郑

**Affiliations:** 华中科技大学同济医学院附属同济医院血液科，武汉 430030 Department of Hematology, Tongji Hospital, Tongji Medical College, Huazhong University of Science and Technology, Wuhan 430030, China

患者，男，59岁，因“双下肢乏力半年，加重1个月，嗜睡伴低热10余天”于2015年4月入院。查体：嗜睡，言语含糊，反应迟钝，有时不能正确对答，不能完全配合检查。双侧腋窝可触及数个米粒大小淋巴结。腹软，膨隆，肝脏肋缘下未及，脾脏触诊肋缘下3 cm，脾缘在脐水平面以上（中度肿大）。颈强直，双侧克氏征（+），右侧病理征可疑阳性，四肢肌力正常。血常规：WBC 9.35×10^9^/L，HGB 112.7 g/L，PLT 111.46×10^9^/L；血钙3.92 mmol/L；LDH 2190 U/L；尿素15.64 mmol/L；肌酐193 µmol/L；尿酸593 µmol/L；碳酸氢根26.9 mmol/L；估算肾小球滤过率31.1 ml·min^−1^·1.73 m^−2^。头部磁共振成像：双侧额顶叶皮层下、半卵圆中心、侧脑室旁多发缺血变性灶；中脑及脑桥右后方静脉瘤，右侧乙状窦与颈内静脉交界处管腔闭塞可能，右侧下颌后静脉较对侧增粗。骨髓细胞形态学：三系增生减低骨髓象；骨髓流式细胞术结果：约7.2％细胞考虑为单克隆性异常B淋巴细胞可能性大，部分表达CD7，Ki-67阳性率50％，弥漫大B细胞淋巴瘤（DLBCL）不能除外。骨髓活检：骨髓增生极度活跃（100％），幼稚细胞弥漫性增生，胞体较大，胞质量中等，胞核圆形或不规则，染色质细致，核仁明显。粒系、红系少见。全片未找到巨核细胞。纤维组织弥漫性增生。免疫组化染色：TdT（−），CD79α（+），CD20（+），CD10（−），bcl-2（+），bcl-6（+），MUM-1（+），CD3（−）；Ki-67阳性指数30％。考虑DLBCL（非生发中心型）侵犯骨髓。淋巴结抗原受体基因重排：检测到IGHA、IGHB、IGHD、IGKA及IGKB单克隆性重排基因片段。腋窝淋巴结活检组织流式细胞术：约68％细胞考虑为单克隆性异常大B淋巴细胞可能性大，部分异常表达CD7，Ki-67阳性率70％，DLBCL不能除外。2015年5月腋窝淋巴结活检病理提示：（左腋窝）淋巴结，符合DLBCL。免疫组化：CD79α（+），CD20（+），PAX-5（+），CD10（−），MUM-1（+），Bcl-6（+），bcl-2（+），CD21（−），Ki-67阳性指数90％，CD43（+），CD3（−），CD5（−），CD2（−）。活检组织细胞染色体分析：核型诊断：49，Y，add（X）（p22），+3，+3，t（1;3）（p32;q27），t（1;11）（p11;q11），+18[10]。淋巴结FISH结果提示：考虑患者存在涉及BCL6位点的染色体易位及扩增，BCL2扩增；淋巴结活检组织标本二代测序结果提示：检出MYD88 p.H269_K271del，TET2 p.F868L，EZH2 p.Y646N，CARD11 p.A581T，MLL3 p.T316S，MLL2 p.Q3499K突变。患者确诊为DLBCL ⅣB期，IPI评分3分。

患者入院后症状体征考虑为肾功能不全、高钙血症所致神经症状，行连续性静脉-静脉血液滤过治疗2次，患者神经症状明显缓解。患者于2015年5月行miniRCHOP方案（长春地辛3 mg，第1天；吡柔比星60 mg，第1天；环磷酰胺900 mg，第1天；地塞米松10 mg，第1～5天；利妥昔单抗600 mg，第6天）化疗2个周期，RCHOP方案（长春地辛4 mg，第1天；吡柔比星90 mg，第1天；环磷酰胺1200 mg，第1天；地塞米松15 mg，第1～5天；利妥昔单抗600 mg，第6天）化疗6个周期。患者化疗后乏力、嗜睡、低热症状完全消退，中期及疗程结束PET-CT疗效评价均为完全缓解。患者规律行利妥昔单抗（375 mg/m^2^）单药维持治疗2年，期间患者一般情况可，无明显症状；2017年9月患者再次出现乏力、盗汗症状，入院复查，骨髓细胞形态学结果示：增生重度减低，粒∶红＝6.9∶1；粒系比例偏高，占69％，以成熟阶段为主。红系受抑，占1％，形态学无异常。淋巴细胞比例偏高，占22％，无异常。血小板散在，小簇易见，未找到巨核细胞。诊断意见：骨髓增生重度减低。复查PET-CT提示颈部双侧、纵隔多处、左侧冈下肌、左侧胸大肌胸小肌间隙多处淋巴结，腹膜后多处淋巴结增大，代谢不均匀增高，考虑淋巴瘤复发。鉴于患者当前疾病属于晚期复发状态（无事件生存期大于24个月），二线化疗的方案选择在原敏感化疗方案（RCHOP）的基础上联合来那度胺（25 mg，隔日1次）及西达本胺（5 mg，每日1次）口服治疗。2017年11月予R2-CHOP+西达本胺方案挽救化疗共4个周期。2018年2月PET-CT回报：纵隔及浅表淋巴结肿大均完全消退。患者以西达本胺5 mg每日1次联合来那度胺25 mg隔日1次维持治疗，截至末次随访（2020年6月），患者多次复诊病情均保持稳定。

